# Preliminary Outcomes of Different Tactics of Ureteral Stent Placement in Patients with Ureteral Stricture Undergoing Balloon Dilatation: Experience from a Large-Scale Center

**DOI:** 10.3389/fsurg.2022.847604

**Published:** 2022-05-16

**Authors:** Xiao Hu, Dechao Feng, Xin Wei

**Affiliations:** ^1^ Department of Urology, Institute of Urology, West China Hospital, Sichuan University, Chengdu, China

**Keywords:** ureteral stricture, balloon dilatation, single ureteral stent, double ureteral stents, triple ureteral stents

## Abstract

**Purpose:**

Our aim is to demonstrate the optimal number of ureteral stent placements in patients with a ureteral stricture (US) after balloon dilatation (BD).

**Methods:**

A retrospective analysis of 213 patients who underwent BD from 2011 to 2019 was conducted. All statistical analyses were completed by software SPSS 25.0.

**Results:**

Of the patients enrolled, 119 were males and 94 were females. The average age was 44.71 years. One month after stent removal, the overall success rate of ureteral stent placement was 76.99%, and the success rates of single, double, and triple stent groups were 81.7%, 70.3%, and 79.3%, respectively. Six months after stent removal, the overall success rate was 61.9%, and the success rates of the three groups were 61.7%, 52.7%, and 74.1%, respectively. Twelve months after stent removal, the overall success rate was 55.9%, and the success rates of the three groups were 51.9%, 48.6%, and 70.7%, respectively. During indwelling of the stents, the proportions of severe bladder irritation symptoms in the three groups were 13.6%, 16.2%, and 20.7%, respectively. Multivariate analysis indicated the length of US and the time and number of ureteral stent placements were independent risk factors of the treatment effect at 6 months and 12 months after stent removal. Patients in the triple stent group had a better prognosis when compared to those in the single or double stent group.

**Conclusion:**

The long-term effect of three stents was better than that of single and double stents, but the success rate of treatment reduced gradually over time.

## Introduction

Ureteral stricture (US) is one of the most commonly encountered problems in the clinical practice of urology, and ureteral reconstruction remains a challenge in the field of reconstructive urology. The US is a common sequela after endoscopic procedures for urinary stones and invasive diagnostic, with an incidence of approximately 3.5% (1). Besides, iatrogenic urinary tract injuries are frequent in pelvic surgeries, like obstetrical and gynecologic surgery, which might contribute to US (2). An increasing number of the US has been observed due to increased endoscopic procedures for kidney and ureteral stone treatment, radiation therapy, and pelvic surgery (3).

Conservative treatment of the US is highly associated with hydronephrosis, urinary tract infection, and deterioration of renal function (4). Conventional open approaches are limited by the risk of larger trauma, bleeding, restricture, longer operation time, and length of stay. With the development of endoscopic techniques, such as incision through endoscopy and balloon dilatation (BD), endoscopic treatment for the US might serve as an alternative to open surgery. The success rate of BD reported in the literature was about 13%−80%, but US might recur over time (5, 6). A study reported that endoscopic BD has a high success rate in the treatment of benign USs, but some points remain controversial such as balloon type, dilatation pressure, expansion number, postoperative ureteral stent type, and stent retention time for the BD technique (7). Another treatment such as laser endoureterotomy provides favorable results, and double ureteral stents benefit more than a single stent in the long-term patency rate (8). Endourological therapy is a cost-effective and minimally invasive method for the treatment of benign short-segment USs (<2 cm). Thus, we proposed whether the number of ureteral stent placements (USPs) exerts an impact on the effect of BD.

## Methods

### Study Design

The study was conducted according to the Declaration of Helsinki (as revised in 2013). A retrospective analysis of 213 patients who underwent BD in our hospital from 2011 to 2019 was conducted. Patients were eligible if they met the following criteria: (1) age ≥18 years; (2) the US was diagnosed by retrograde pyelography; and (3) the diameter of the US was less than 2 mm, which was about double the width of a guidewire. Exclusion criteria are the following: (1) ureteral atresia; (2) the length of US was more than 5 cm; (3) the guidewire or balloon failed to pass through the stricture segment; and (4) the US was derived from exogenous compression, uncontrolled cancers, and oncologic invasion.

### Procedures

Retrograde BD was performed transurethrally with lithotomy position under general anesthesia. Two hydrophilic coated guidewires were passed through the US under the vision of a ureteroscopy, and subsequently, a balloon dilator (21F, Bard Medical, Covington, GA, United States) was placed over the narrowed segment. We confirmed the position of the guidewire or balloon dilator with the C-arm of an X-ray machine. An iodine contrast agent was injected into the high-pressure balloon, and the pressure was maintained at 25 atmospheres for several minutes. The definition of expansion completion was that the pressure did not decrease with time and was stable at 25 atmospheres. We would perform ureteropyelography to ensure that the stenosis was fully expanded after dilatation. After the operation, one to three 4.7 Fr or 6 Fr ureteral double J stents (Bard Medical) were indwelled for 1–6 months according to the degree of ureteral dilatation and injury. There was no intraoperative complication that needed further interventions.

### Follow-Up

Color Doppler ultrasound, diuretic renogram, or abdominal computerized tomography was used to assess the hydronephrosis at 1 month after stent removal. The success of the operation is defined as no increase in hydronephrosis and deterioration of renal function after removing the USP. Demographic data, the position and length of the US, time of USP, serum creatinine, and follow-up time were collected to compare the effect of different numbers of USPs.

### Statistical Analysis

Quantitative data with normal distribution were described as mean ± standard deviation; otherwise, median and interquartile range were used. When the quantitative data were normally distributed and the variance was homogeneous, analysis of variance or independent sample *t*-test was used for comparison between groups, and the SNK-q test was used for pairwise comparison between groups. Kruskal–Wallis rank-sum test was used between different stent groups when the data did not follow a normal distribution or the variance was uneven. The Mann–Whitney rank-sum test was used between the two groups. The categorical variables were expressed as proportion and percentage, and the comparison between groups was performed by the *χ*^2^ test. The test level of comparison between the two groups was *α* = 0.05. All statistical analyses were completed by software SPSS 25.0.

## Results

A total of 213 individuals met the criteria for inclusion, including 119 males and 94 females. The average age was 44.71 years. The average length of the US was 1.52 cm, and the average time of indwelling stent was 4.78 months. Besides, the patients were followed up for at least 12 months. The numbers of patients indwelling a single stent, double stents, and triple stents were 81, 74, and 58, respectively. The causes of US included stone-related operations (ureteroscopic lithotripsy or ureterolithotomy and extracorporeal lithotripsy, 59.6%), ureteropelvic junction obstruction (12.7%), other surgical injuries (10.8%), and unclear etiology (16.9%).

The overall success rate of BD was 51.1% until the last follow-up. The patients who failed to BD underwent surgical ureteral reconstruction, nephrostomy, placement of allium metal stent, and even nephrectomy. One month after stent removal, the overall success rate of USP was 76.99%, and the success rates of single, double, and triple stent groups were 81.65%, 70.3%, and 79.3%, respectively. Six months after stent removal, the overall success rate of USP was 61.9%, and the success rates of single, double, and triple stent groups were 61.7%, 52.7%, and 74.1%, respectively. Twelve months after stent removal, the overall success rate of USP was 55.9%, and the success rates of single, double, and triple stent groups were 51.9%, 48.6%, and 70.7%, respectively. We divided the bladder irritation symptoms into three levels after placing the stents: mild, moderate, and severe. We evaluated the irritation of the stent to the bladder based on the overactive bladder symptom score (OABSS). If OABSS ≤ 5, or symptoms are not sufficient to meet the OAB diagnostic criteria, we consider that the stent has mild irritation to the bladder; if OABSS ranges from 6 to 11, we classify it moderate; if OABSS ≥ 12, we classify it severe. The proportions of severe bladder irritation symptoms in the three groups were 13.6%, 16.2%, and 20.7%, respectively. The basic characteristics of the patients included in this study are summarized in Table 1.

**Table 1:** Baseline characteristics of enrolled patients in this study.

Features	Single stent	Double stents	Triple stents	X/F	*p-*value
Male/female	31/50	40/34	23/35	4.555	0.103
Age (years)	41.44 ± 13.56	46.81 ± 14.09*	46.59 ± 12.32*	3.870	0.022
BMI (kg/m^2^)	22.81 ± 3.04	23.34 ± 3.12	23.40 ± 2.87	0.864	0.423
SCR (umol/L)	80 (67, 99)	78 (67, 88)	84 (65, 100)	0.641	0.726
Length of US (cm)	1 (0.5, 1)	1 (1, 2)	1.5 (1, 2)*	10.035	0.007
Side				3.002	0.223
Left	49	35	29		
Right	32	39	29		
1 m after UTR				2.985	0.225
Valid	66	52	46		
Invalid	15	22	12		
6 m after UTR				6.343	0.042
Valid	50	39**	43		
Invalid	31	35	15		
12 m after UTR				7.262	0.026
Valid	42	36**	41		
Invalid	39	38	17		
Last follow-up				8.444	0.015
Valid	38	32**	39		
Invalid	43	42	19		
Level of BIS				6.370	0.173
Mild	51	45	25		
Moderate	19	17	21		
Severe	11	12	12		

*SCR, serum creatinine; US, ureteral stricture; UTR, ureteral stent removal; IS, bladder irritation symptoms.*

* Compared to a single stent, p < 0.05; **Compared to three stents, p < 0.05.

Supplementary Table S1 and Table 2, respectively, showed the risk factors related to the prognosis of patients, and the multivariate analysis of surgical success at 1 month after stent removal was negatively correlated with the length of the US and the time of USP but not with the number of USP. However, the length of the US, the time of USP, and the number of USPs were independent risk factors of the treatment effect at 6 months (Supplementary Table S2 and Table 3) and 12 months (Supplementary Table S3 and Table 4) after stent removal. Furthermore, patients in the triple stent group had a better prognosis when compared to those in the single or double stent group.

**Table 2:** Risk factors related to the prognosis of patients at 1 month after stent removal using multivariate analysis.

Variables	Regression coefficients	Standard error	Wald test	*p-*value	OR	95% CI
Length of US	−0.474	0.144	10.810	0.001	0.622	0.469–0.826
Time of USP	−0.232	0.082	8.230	0.004	0.791	0.674–0.929
Single stent			1.109	0.574		
Double stents	0.056	0.442	0.016	0.899	1.058	0.444–2.517
Triple stents	0.452	0.476	0.904	0.342	1.5572	0.619–3.994
Constant	3.029	0.511	35.091	<0.001	20.680	

*US, ureteral stricture; USP, ureteral stent placement; OR, odds ratio; CI, confidence interval.*

**Table 3:** Risk factors related to the prognosis of patients at 6 months after stent removal using multivariate analysis.

Variables	Regression coefficients	Standard error	Wald test	*p-*value	OR	95% CI
Length of US	−0.372	0.135	7.612	0.006	0.689	0.529–0.898
Time of USP	−0.162	0.072	5.036	0.025	0.851	0.739–0.980
Single stent			7.545	0.023		
Double stents	0.112	0.373	0.091	0.763	1.119	0.539–2. 323
Triple stents	1.050	0.416	6.355	0.012	2.857	1.263–6.463
Constant	1.528	0.400	14.599	<0.001	4.610	

*US, ureteral stricture; USP, ureteral stent placement; OR, odds ratio; CI, confidence interval.*

**Table 4:** Risk factors related to the prognosis of patients at 12 months after stent removal using multivariate analysis.

Variables	Regression coefficients	Standard error	Wald test	*p-*value	OR	95% CI
Length of US	−0.373	0.138	7.291	0.007	0.689	0.525–0.903
Time of USP	−0.145	0.071	4.138	0.042	0.865	0.752–0.995
Single stent			10.357	0.006		
Double stents	0.327	0.369	0.785	0.376	1.387	0.673–2. 859
Triple stents	1.265	0.405	9.741	0.002	3.542	1.601–7.839
Constant	1.043	0.385	7.324	0.007	2.838	

*US, ureteral stricture; USP, ureteral stent placement; OR, odds ratio; CI, confidence interval.*

## Discussion

Regarding the number of stents placed in patients, at the early stage of starting BD treatment in our hospital, only one stent was placed in the ureter of all patients. In the middle stage, two stents were placed in all patients after BD. In the last 4 years, the placement of the three-bracket strategy was widely applied.

The use of stents after ureteral dilation or incision might contribute to ureteral healing, prevention of urine extravasation, and avoiding restenosis (9). However, the management of optimal USP following endourologic treatment of US remains controversial (10). It is noticeable that there is no positive correlation between the stent size and the therapeutic effect of US. Several studies indicated that the use of a 14F stent provided no advantage over the use of a smaller, more easily positioned 7F stent (11, 12). Currently, evidence assessing the effect of the number of USP on US after BD is still deficient.

In most cases, the number of stents depends on the preference of surgeons and the degree of the US. In 1998, Liu et al. (13) found the use of two ipsilateral ureteral stents was beneficial in relieving flank pain and persistent azotemia in four patients who failed the single stent due to ureteral obstruction secondary to non-urinary tract malignancies. Subsequently, several clinical studies indicated that placing two parallel stents simultaneously provides a more favorable effect than a single stent after endoureteral treatment of the ureter (14–16). Endourological treatments have been tried for benign short-segment USs (<2 cm). Thus, increased stents had potential advantages in drainage effect, and a better drainage effect is conducive to local tissue healing. Our study observed that three parallel stents were better than a single stent and double stents, but no significant difference was detected between the single stent and double stents. We believed that the urine drainage of the ureteral stent did not depend on the lumen of the stent but on the peritubular space. The increased stiffness of three stents reduced kinking and luminal compression, and the potential space between the stents likely preserved flow around as well as through them (13). Additionally, some researchers proposed that the relative motion between ureteral stents might provide a continuous dilation effect and preclude the formation of stenosis (17). However, plethoric stents could lead to local ischemia in the US segment, affecting tissue healing. Despite the obvious success rate of three stents over single or double stents, further studies on the effect of the number of stents on local tissue are warranted.

The length of the US is also an essential factor for urologists. Some studies showed that the success rate of BD for a benign US < 2 cm was higher (18, 19), and some studies had more strict requirements on the length of the US. They believed that a US < 1.5 cm had a higher success rate of BD, and open surgery should be considered for longer US (20). We suggested that the short stricture is more beneficial to increasing the success rate of BD according to our results. There is also no uniform standard for the placement time of the stent. Some complications follow indwelling stents such as low back pain, hematuria, bladder irritation, and so on. Long-term indwelling stents can cause local inflammation, promote the proliferation of scar tissue, and affect incision healing (21). Our studies showed that the indwelling time of stents in the effective group was significantly shorter than that in the ineffective group. How to choose the placement time needs further research. The reported study indicated that the success rate of BD gradually decreased over time (22). This trend was consistent with our study, but the decline of multiple stents was slower.

In the results, there was no significant difference in prognosis between patients with a single stent and double stents, but prognosis in the triple stent group was conspicuously different from the other two groups. We guess the supporting effect of one and two stents was not enough to be reflected in patients after BD in the ureter because the injury or healing method of BD may be different from endoureterotomy and discontinuous anastomosis in the ureter.

There is no denying that our study had the following limitation. First, the inherent limitations of a retrospective study and limited sample size preclude us from making a definite and robust conclusion. Second, complications and quality of life are also absent. Besides, whether complications, like urinary tract infection, could exert a significant impact on renal function could not be further evaluated. Despite these limitations, our study does provide some evidence for the management of such patients.

## Conclusions

The long-term effect of three stents is better than that of single and double stents, but the success rate of treatment reduces gradually over time. Further large, well-designed trials are warranted to confirm our findings.

## Data Availability

The original contributions presented in the study are included in the article/Supplementary Material; further inquiries can be directed to the corresponding author/s.
