# Supplementary material for: Preliminary Outcomes of Different Tactics of Ureteral Stent Placement in Patients with Ureteral Stricture Undergoing Balloon Dilatation: Experience from a Large-Scale Center
Source: Front Surg. 2022 May 16;9:847604. doi: 10.3389/fsurg.2022.847604 (PMC9149213; doi:10.3389/fsurg.2022.847604)
Supplement: Supplementary file 1 [file Table_1_v1.docx]

Supplementary table 1. The risk factors related to the prognosis of patients at one month after stent removal using univariate analysis.

| Variables | Valid | Invalid | χ^2^/F | P value |
| --- | --- | --- | --- | --- |
| Male/Female | 89/75 | 30/19 | 0.740 | 0.390 |
| Age(years) | 43.84±13.29 | 47.61±14.37 | 1.710 | 0.089 |
| Length of US (cm) | 1(0.8,1.8) | 1.1(0.9,3.0) |  | 0.034 |
| Time of USP (month) | 4(3,6) | 6(6,6) | 2.081 | 0.001 |
| Side |  |  | 0.424 | 0.515 |
| Left | 89 | 24 |  |  |
| Right | 75 | 25 |  |  |
| Position of US |  |  | 0.744 | 0.689 |
| Upper | 85 | 24 |  |  |
| Middle | 22 | 9 |  |  |
| Lower | 57 | 16 |  |  |
| Number of Stents |  |  | 2.985 | 0.225 |
| Single | 66 | 15 |  |  |
| Double | 52 | 22 |  |  |
| Triple | 46 | 12 |  |  |

US= ureteral stricture; USP: ureteral stent placement.

Supplementary table 2. The risk factors related to the prognosis of patients at six months after stent removal using univariate analysis.

| Variables | Valid | Invalid | χ2/F | P |
| --- | --- | --- | --- | --- |
| Male/Female | 70/62 | 49/32 | 1.134 | 0.287 |
| Age(years) | 44.70±13.45 | 44.73±13.94 | 0.016 | 0.987 |
| Length of US (cm) | 1(0.8,1.8) | 1(0.8,2.5) |  | 0.136 |
| Time of USP (month) | 4(3,6) | 6(3,6) |  | 0.005 |
| Side |  |  | <0.001 | 0.994 |
| Left | 70 | 43 |  |  |
| Right | 62 | 38 |  |  |
| Position of US |  |  | 3.257 | 0.196 |
| Upper | 68 | 41 |  |  |
| Middle | 15 | 16 |  |  |
| Lower | 49 | 24 |  |  |
| Number of Stents |  |  | **6.343** | **0.042** |
| Single | 50 | 31 |  |  |
| Double | 39 | 35 | * |  |
| Triple | 43 | 15 |  |  |

US= ureteral stricture; USP: ureteral stent placement.

Supplementary table 3. The risk factors related to the prognosis of patients at twelve months after stent removal using univariate analysis.

| Variables | Valid | Invalid | χ2/F | P |
| --- | --- | --- | --- | --- |
| Male/Female | 62/57 | 57/37 | 1.553 | 0.213 |
| Age(years) | 44.65±13.45 | 44.79±13.87 | 0.074 | 0.941 |
| Length of US (cm) | 1(0.8,1.8) | 1(0.8,2.4) |  | 0.201 |
| Time of USP (month) | 4(3,6) | 6(3,6) |  | 0.040 |
| Side |  |  | 0.001 | 0.971 |
| Left | 63 | 50 |  |  |
| Right | 56 | 44 |  |  |
| Position of US |  |  | 3.429 | 0.180 |
| Upper | 61 | 48 |  |  |
| Middle | 13 | 18 |  |  |
| Lower | 45 | 28 |  |  |
| Number of Stents |  |  | 7.262 | **0.026** |
| Single | 42 | 39 |  |  |
| Double | 36 | 38 | * |  |
| Triple | 41 | 17 |  |  |

US= ureteral stricture; USP: ureteral stent placement.
